# Goal-Guided Graph Attention Network with Interactive State Refinement for Multi-Agent Trajectory Prediction

**DOI:** 10.3390/s24072065

**Published:** 2024-03-23

**Authors:** Jianghang Wu, Senyao Qiao, Haocheng Li, Boyu Sun, Fei Gao, Hongyu Hu, Rui Zhao

**Affiliations:** 1 College of Automotive Engineering, Jilin University, Changchun 130025, China; wujh1521@mails.jlu.edu.cn (J.W.); qiaosy1521@mails.jlu.edu.cn (S.Q.);; 2 State Key Laboratory of Automotive Simulation and Control, Jilin University, Changchun 130025, China

**Keywords:** autonomous driving, trajectory prediction, attention mechanism, scene feature map

## Abstract

The accurate prediction of the future trajectories of traffic participants is crucial for enhancing the safety and decision-making capabilities of autonomous vehicles. Modeling social interactions among agents and revealing the inherent relationships is crucial for accurate trajectory prediction. In this context, we propose a goal-guided and interaction-aware state refinement graph attention network (SRGAT) for multi-agent trajectory prediction. This model effectively integrates high-precision map data and dynamic traffic states and captures long-term temporal dependencies through the Transformer network. Based on these dependencies, it generates multiple potential goals and Points of Interest (POIs). Through its dual-branch, multimodal prediction approach, the model not only proposes various plausible future trajectories associated with these POIs, but also rigorously assesses the confidence levels of each trajectory. This goal-oriented strategy enables SRGAT to accurately predict the future movement trajectories of other vehicles in complex traffic scenarios. Tested on the Argoverse and nuScenes datasets, SRGAT surpasses existing algorithms in key performance metrics by adeptly integrating past trajectories and current context. This goal-guided approach not only enhances long-term prediction accuracy, but also ensures its reliability, demonstrating a significant advancement in trajectory forecasting.

## 1. Introduction

Trajectory prediction stands as one of the most challenging aspects of autonomous driving, requiring the model to accurately predict the trajectories of traffic participants (e.g., vehicles, pedestrians, cyclists, etc.) surrounding the autonomous vehicle [1]. This process involves numerous potential variables. The emergence of high-definition maps (HD maps) and datasets from sensors has propelled research in this field [2,3]. Combining map information and sensor data has become an effective strategy to improve prediction accuracy, albeit at the cost of increasing the complexity of the prediction process [4]. Effectively leveraging this information poses a central challenge in the field of trajectory prediction.

To achieve rapid and accurate trajectory prediction, motion models based on vehicle physics characteristics have been widely adopted [5,6]. These methods primarily rely on the vehicle’s past motion states (position, velocity, acceleration, etc.) and employ filtering and optimization techniques to predict future maneuver strategies, such as Kalman filters (KFs), Dynamic Bayesian Networks (DBNs), and Hidden Markov Models (HMMs) [7,8,9]. In comparison to models based on vehicle kinematics, those based on deep learning are increasingly gaining popularity [10,11]. They excel in extracting hidden dependencies from contextual time steps, and are particularly adept at capturing long-term features in trajectories. Alahi et al. [12] pioneered the use of Social-LSTM, incorporating a social pooling layer to explore interactions among pedestrians. Inspired by this research, many researchers have started deploying similar model architectures to model social interactions among agents. Nachiket et al. [13] introduced convolutional operations in the social pooling layer, achieving promising experimental results in predicting vehicle trajectories. Sheng et al. [14] proposed a Graph-based Spatiotemporal Convolutional Network (GSTCN) that utilizes graph convolutional networks to handle spatial interactions. Given the breakthroughs of Transformers [15] in natural language processing and their wide-spread adoption for predicting agent behavior due to their long-term predictive capabilities [16,17], they efficiently address the memory problem of handling long sequences with attention mechanisms. The model can directly associate the entirety of input data sequences and context vectors, rather than being limited to the association with the last hidden states [18]. Syed et al. [19] introduced the Spatiotemporal Graph Transformer (STGT) model, which uses CNN models for processing environmental image features and employs Transformers for sequence prediction. Mercat et al. [20], by introducing self-attention mechanisms considering interactions between vehicles, successfully achieved trajectory prediction for multiple vehicle agents. Roger et al. [21] proposed the AutoBots model, which, through the use of social multi-head self-attention (MHSA) modules, efficiently performs single-pass forward inference for the entire future scene, demonstrating high performance in handling complex traffic scenarios with multi-agent interactions. The adoption of deep learning models like Transformers and MHSA modules has significantly advanced multi-agent trajectory prediction in complex traffic scenarios. However, challenges such as the need for real-time adaptability, integrating dynamic environmental conditions, and reducing model complexity without sacrificing accuracy, remain areas ripe for future research.

With the advent of large-scale datasets, the introduction of high-definition maps (HD maps) and sensor data has brought about breakthroughs in the field of trajectory prediction [22,23]. In the past, predicting vehicle trajectories primarily relied on the physical properties of vehicles, such as historical trajectories, speed, acceleration, and the relative distances to surrounding vehicles. While these methods perform well in simple traffic environments, the trajectories of vehicles in complex road scenarios are influenced not only by surrounding vehicles (SVs), but also by lane guidance and spatial constraints imposed by road boundaries. Therefore, the inclusion of HD maps around the vehicle and its sensor data in the scope of trajectory prediction has become increasingly necessary.

To enable neural networks to handle HD maps, some studies rasterize map data and then apply convolutional neural networks (CNNs) to extract features from it [24,25]. Casas et al. [26] utilized a CNN detector to extract features from rasterized maps. Hong et al. [27] employed high-precision 3D perception and detailed semantic environmental maps. They encoded semantic information through spatial grid encoding and used deep convolutional models to integrate complex scene contexts. Rasterizing map data aims to obtain long-range information along the lane direction, requiring a relatively large perception field, which may lead to significant computational resource waste [28]. Additionally, rasterization processing may result in information loss. Simply inputting map data into the model might not effectively capture complex information such as road structures, traffic signs, signals, etc. Therefore, there is a need for a deeper integration of vehicle trajectories and maps to address these challenges.

Another option is to use vectorized map features. VectorNet [29] vectorizes HD maps and agents’ trajectories, employing graph neural networks to depict interactions between traffic participants and road environment, as well as interactions among the participants themselves. It can effectively capture complex interactions between traffic participants and structural information in the road environment, avoiding information loss introduced by rasterization, thus providing a more comprehensive map representation. MTR++ [30] uses a local self-attention mechanism to capture essential local structural information in vectorized road maps. This enables the system to more accurately understand and predict the future movements of multiple agents in complex traffic environments. Liang et al. proposed LaneGCN [31], which constructs a map node graph and uses a multi-step graph neural network to encode the map, considering the road’s topological structure. This approach clarifies the interactions among traffic participants and more accurately represents their connection to map structures. However, due to the diverse ways in which actors move, fixed-size strides cannot effectively model distant-related map features, thereby limiting predictive performance.

To address the challenges mentioned above, this paper introduces an innovative trajectory prediction framework, which we refer to as “SRGAT” (a goal-guided and interaction-aware state refinement graph attention network), building upon the LaneGCN [31] baseline proposed by Liang et al. This framework integrates high-definition maps and vehicle dynamics, employing lane graph convolution operators to capture complex traffic scenarios. The social encoding component combines 1D CNN with FPN to extract interactions between vehicles, utilizing a multi-head self-attention mechanism to further understand social relationships. The model estimates potential target points for vehicles and refines predictions by incorporating deep contextual information, enhancing prediction accuracy. The decoder utilizes a recursive feed-forward network and multi-head attention decoder layers to iteratively predict multimodal future trajectories. Each trajectory is based on a learnable seed parameter matrix and comes with an associated confidence score, allowing the model to consider probability distributions. By integrating high-definition maps and agent state information, dynamic interaction features from the social encoder, as well as advanced target estimation and trajectory generation strategies, our model ensures high-precision trajectory prediction in complex traffic situations.

The contributions of this paper can be summarized as follows:

1. Leveraging prior research, our study introduces SRGAT, a cutting-edge trajectory prediction framework that innovatively merges HD maps with dynamic vehicle data. This method not only addresses the challenge of fixed stride by adapting to vehicle context and environmental objectives, but also comprehensively evaluates the road environment’s influence on trajectory forecasts.
2. Our model significantly boosts the accuracy of trajectory predictions in intricate traffic scenarios by exploiting HD maps’ spatial constraints and vehicles’ dynamic states, effectively addressing the challenge of dynamic goal estimation.
3. By introducing a dual-branch multimodal prediction architecture that generates multiple potential future trajectories and assigns a confidence score to each, the model’s accuracy and diversity in trajectory prediction in complex traffic situations are significantly enhanced. It increases both the accuracy and variety of the predictions.
4. We conducted evaluations of the proposed model on both Argoverse and nuScenes datasets and engaged in a detailed comparison with the current state-of-the-art methods. The results demonstrate that our model exhibits substantial performance improvements over these methods across a range of critical performance metrics.

The remainder of this paper is organized as follows: Section 2 defines the problem of trajectory prediction. Section 3 details the data processing methods. Section 4 describes the network structure of the algorithm and presents the training details. Section 5 discusses the experimental setup, results, and comparisons with existing methods. Finally, conclusions are drawn in Section 6.

## 2. Problem Formulation

We assume that by observing traffic participants and their environment, it is possible to capture precise historical motion paths and high-resolution map data in a two-dimensional coordinate system. Specifically, for the *i*th agent in all *n* traffic participants, we can collect a series of state observations $S_{\mathrm{obs}}^{i}=\left[s_{-T+1}^{i},s_{-T+2}^{i},\ldots ,s_{0}^{i}\right],i\in \left[0,n-1\right]$ within a certain time frame {−T+1,−T+2,…,0}, where $s_{t}^{i}=\left(x_{t}^{i},y_{t}^{i},v_{x,t}^{i},v_{y,t}^{i},a^{i},o_{t}^{i}\right)$, consisting of the x,y coordinate, velocity, agent type and orientation in a global Cartesian coordinate as features at time step *t*. A corresponding high-definition map m was added to establish a complete scene. The scene information set m={Y,A} can be divided into a lane node feature matrix Y and an adjacency matrix set ${\left\{A_{i}\right\}}_{i\in \{\mathrm{pre},\mathrm{suc},\mathrm{left},\mathrm{right}\}}$. The node matrix $Y=\left(x^{j},y^{j},heading^{j},turn^{j},traf^{j},intersect^{j}\right),j\in N_{node}$ represents the lane geometry feature and adjacency matrix represents the topology connections between different nodes. The meaning of each matrix in m will be further explained in Section 3. The goal of this research is to predict the agent’s future states $S_{\mathrm{out}}=\left[s_{1},s_{2},\ldots ,s_{t}\right]$. With the current map, past states of the agent, and the states of other agents known, we aim to define the probability distribution of the agent’s future states, $p(S_{\mathrm{out}}|m,S_{\mathrm{obs}},S_{\mathrm{obs}}^{O})$, where $S_{\mathrm{obs}}^{O}$ indicates the observed states of other agents and $S_{\mathrm{obs}}^{ego}$ stands for ego agents when i=0. Our model offers K modes of potential future trajectory sets ${\left\{\left\{S_{\mathrm{out},k}\right\}\right\}}_{k\in [0,K-1]}={\left\{\left\{\left(S_{k}^{1},S_{k}^{2},\ldots ,S_{k}^{T}\right)\right\}\right\}}_{k\in [0,K-1]}$ for each agent.

## 3. Data Preprocessing

**Figure 1:**
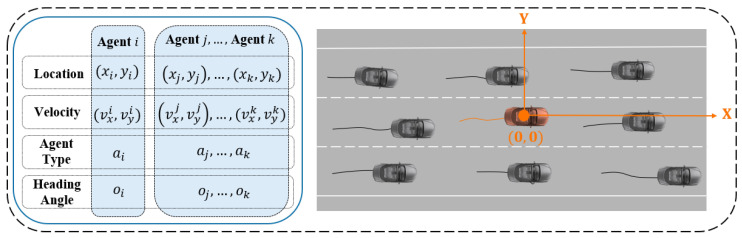
Visualization of data preprocessing for the SRGAT model. The left side presents the structured data inputs for agents. The right side demonstrates the conversion of map metadata into a vectorized road network graph, oriented on a 2D Cartesian plane with the ego vehicle at the center.

In the process of map information preprocessing, we first transform the map metadata from the Argoverse dataset into a vectorized map data representation. This approach primarily represents the map data as a graph structure, aiming to characterize a set of lanes and their connectivity. The course of the roads is represented by the two-dimensional coordinates of discretized road centerlines. To better utilize the relationship between the road and the ego vehicle, we adopt a 2D Cartesian coordinate system with the ego vehicle at the origin and the forward direction as the x-axis. The two-dimensional coordinates of the centerlines serve as the nodes of the graph, forming a series of 2D bird’s eye viewpoints arranged according to lane direction. For any two directly accessible lanes, we define four types of connectivity—predecessor, successor, left neighbor, and right neighbor—and encode these connections as edge information in the graph, as shown in Figure 1.

Specifically, lanes that can be directly accessed are considered neighboring predecessors and successors, while left and right neighbors are defined as the spatially nearest lane nodes on adjacent lanes to the left and right, measured by Euclidean distance. We represent lane nodes with $V\in R^{N\times 2}$(can be seen as part of Y), where $N_{node}$ is the number of lane nodes, and the *i*th row of V represents the BEV coordinates of the *i*th node. We use four adjacency matrices ${\left\{A_{i}\right\}}_{i\in \{\mathrm{pre},\mathrm{suc},\mathrm{left},\mathrm{right}\}}$ to represent the connectivity, where $A_{i}\in R^{N\times N}$. The element in the *j*th row and *k*th column of $A_{i}$ is denoted by $A_{i,jk}$. If node *k* is a type *i* neighbor of node *j*, then $A_{i,jk}=1$.

In the preprocessing of trajectory information, participants located within a specific Euclidean distance from the ego vehicle (which is set to 10 m in our experiments) are exclusively considered to reduce the algorithm’s complexity. To better leverage the relationship between participants and the road (especially the road centerlines) in the environment, all participant trajectory position information need to be represented in terms of relative position to nearby road centerlines. The input for participant *n* is a series of relative displacements: $\Delta v_{n}^{t}=v_{n}^{t}-v_{n}^{t-1}$, where $v_{n}^{t}$ represents the state vector, taking into account the participant’s type (pedestrian, vehicle, cyclist). This representation method further utilizes the symmetry of the problem and avoids the disruption of learning due to changes in the ego vehicle’s absolute position coordinates during subsequent training.

## 4. Structure of SRGAT Model

### 4.1. Model Framework

**Figure 2:**
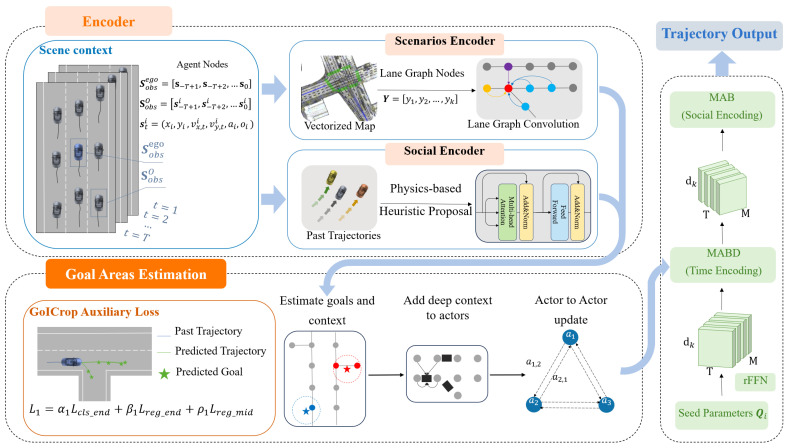
Schematic of the SRGAT model for vehicle trajectory prediction. The Encoder phase assesses the scene via agent nodes and transforms lane graphs into vectorized maps. Subsequent processing by the scenario and social encoders refines these data. Goal Areas Estimation then projects potential destinations, incorporating context into actor dynamics with the help of auxiliary losses. The culmination is the Trajectory Output, which synthesizes predictions using Multi-Agent Behavior (MAB) and Time Encoding (MABD) informed by initial seed parameters.

This section presents the network architecture utilized for trajectory prediction, as illustrated in Figure 2. It comprises three main components: the Encoder, Goal Areas Estimation, and Trajectory Decoding and Generation. The Encoder encompasses a scenario encoder and a social encoder. The scenario encoder processes high-precision map data, converting them into relevant information such as road layouts and geometric shapes. The social encoder processes the historical trajectories of vehicles, using a one-dimensional convolutional network to extract interaction features, and employing Transformer layers to assimilate behavioral patterns of different vehicles. The Goal Area Estimation predicts vehicle destinations using Transformer encoder embeddings to improve precision. The GoICrop mechanism refines this by focusing on crucial trajectory segments, reducing the impact of poor anchor generation on stability. The Trajectory Decoder then constructs the predicted paths, integrating these estimates with dynamic behavior for accurate forecasts.

### 4.2. Encoder

In this section, the main task is to acquire the historical states and environmental features of traffic participants within a scene, learn structured map representations, and then fuse the information of traffic participants with HD map data.

#### 4.2.1. Scenario Encoder

To initiate the encoding process of scenarios, we first input lane map data into a graph convolutional network for feature extraction. It considers the size, orientation, and position of the lane graph nodes when encoding their information, leading to a defined set of features for these nodes:

(1) $$Y=[y_{1},y_{2},\ldots ,y_{k}]$$

(2) $$y_{i}={\mathrm{MLP}}_{\mathrm{shape}}\left(v_{i}^{\mathrm{end}}-v_{i}^{\mathrm{start}}\right)+{\mathrm{MLP}}_{\mathrm{loc}}\left(v_{i}\right)$$

where MLP denotes a multi-layer perceptron with subscripts indicating shape and location. Additionally, $v_{i}$ represents the position of the *i*th lane node, $v_{i}^{\mathrm{start}}$ and $v_{i}^{\mathrm{end}}$ are the coordinates of the start and end points of node *i*, respectively, and $y_{i}$ is the feature of the *i*th graph node. To implicitly capture the directional information carried between graph nodes and seize the long-distance road information relied upon by vehicles during their journey, we employed the LaneConv operator:

(3) $$F=YW_{0}+\sum_{i\in \{\mathrm{left},\mathrm{right}\}}A_{i}YW_{i}+\sum_{C=1}^{C}\left(A_{\mathrm{pre}}^{k_{c}}YW_{\mathrm{pre},k_{c}}+A_{\mathrm{suc}}^{k_{c}}YW_{\mathrm{suc},k_{c}}\right)$$

where F is the aggregated graph node feature, *C* is the dilation size, and $A_{i}$ and $W_{i}$ are the adjacency matrices and weight matrices corresponding to the *i*th type of connection, respectively. $A_{\mathrm{pre}}$, $A_{\mathrm{suc}}$, $A_{\mathrm{left}}$, and $A_{\mathrm{right}}$ represent connections from a node to its immediately preceding, succeeding, left, and right nodes, respectively. $A_{\mathrm{pre}}^{k_{c}}$ denotes the *k* power of $A_{\mathrm{pre}}$, allowing lane graph node features to propagate information along the lane graph for $k_{c}$ steps, where *k* is a hyperparameter. With the utilization of graph convolutional networks and LaneConv, the scenario encoder effectively captures spatial relationships and directional information between lane nodes, enabling the creation of informative graph node features.

#### 4.2.2. Social Encoder

**Figure 3:**
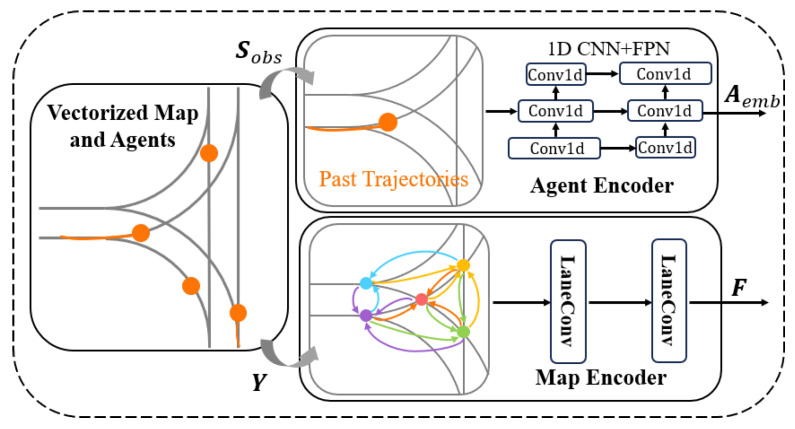
Encoding process of the SRGAT model, capturing past trajectories and environmental features for traffic scenario analysis.

To encode the social interactions among traffic participants, which are crucial for understanding and predicting their behavior, we employ a one-dimensional convolutional neural network (1D CNN). As shown in Figure 3, the network processes the trajectory information of dynamic objects $S_{\mathrm{obs}}$. The 1D CNN architecture consists of multiple scales of convolutional layer groups, each featuring two residual blocks with a stride of 2, enabling the model to capture a wide range of temporal patterns. A feature pyramid network (FPN) [32] is utilized to integrate feature maps of different scales, obtaining the final feature tensor through additional residual blocks. The above process can be expressed as follows:

(4) $$A_{\mathrm{emb}}=\mathrm{FPN}\left(\mathrm{conv}1d\left(S_{\mathrm{obs}}\right)\right)$$

(5) $$\mathrm{FPN}={\mathrm{conv}1d}_{1}\left({\mathrm{conv}1d}_{2}\left({\mathrm{conv}1d}_{3}\left(\right)\right)\right)$$

Each convolutional layer in the network employs a 3×3 kernel size, outputting 128 feature channels, followed by layer normalization and ReLU activation function. This configuration is designed to capture the nuances of dynamic object behavior in detail. After encoding the scene and trajectory information, we relatively obtain a 2D feature matrix $A_{\mathrm{emb}}$ from Formula (4), where each row $A_{\mathrm{emb},i}$ represents the features of the *i* participant, and a 2D feature matrix Y as mentioned above, where each row $Y_{i}$ represents the features of the *i*th lane node. To merge social and environmental information, we utilize the Transformer Fusion Layer. This layer combines the dynamic impact of actors on lanes and the real-time feedback of lanes on actor behavior, subsequently enhancing the interaction between actors and the map through a Transformer encoder. Specifically, we first combine the features of actors $A_{\mathrm{emb},i}$ with those of surrounding lane nodes $Y_{i}$ through a spatial attention mechanism, forming a weighted feature representation $W_{i}$, enriched with each lane node’s characteristics. Then, this module integrates the updated features $Y_{i}^{\prime }$ of lane nodes with the features $A_{\mathrm{emb},i}$ of actor nodes to reflect the real-time impact of lane information on actor behavior. This process not only captures the current traffic state of lane nodes, but also encodes the influence of traffic flow on these lanes. Consequently, it ensures the model’s ability to comprehensively understand and predict the potential behaviors of actors in specific traffic environments.

### 4.3. Goal Areas’ Estimation

To accurately capture and predict the complexity of driving behaviors, it is crucial to avoid oversimplification and accuracy loss in trajectory prediction. We are committed to optimizing our model, ensuring that the predicted trajectories closely match the actual trajectories. After processing through the encoder, the data flow toward the Transformer Fusion Layer, which includes a module for locating target areas. Due to the stochastic and multimodal nature of driving behaviors, we employ multi-target prediction when locating target areas. Initially, we select the most confident target point from the predicted target set as the predicted goal, considering the vehicle’s motion history and current driving environment. Which can be expressed as

(6) $$\mathrm{GP}=\mathrm{FFN}(\mathrm{TransformerEncoder}\left(\mathrm{type}\_\mathrm{encoding}\left(A_{\mathrm{emb}},Y\right)\right))$$

where GP is a 2D matrix in which ${\mathrm{GP}}_{i}$ can be seen as a representation of the likely intended endpoint of the prediction trajectories. Before being processed by the Transformer encoder, $A_{\mathrm{emb}}$ and Y were type-encoded the same way as mentioned in ViLT [33]. This process approximates the vehicle’s destination with the highest probability. By cropping the map to create a target area, we ensure that the vehicle’s final position is more likely to be within this optimized, relatively smaller area. This approach addresses the uncertainty in endpoint prediction and enhances the accuracy of the prediction, making it more likely for the vehicle’s actual position to appear within this smaller area of focus, rather than relying on potentially fluctuating target points.

Based on these predicted target points, we implicitly construct a model of future interactions among actors using the GoICrop [28] technique, which can be expressed as follows:

(7) $${\mathrm{GP}}^{\prime }=\mathrm{GOICrop}\left(\mathrm{GP},Y\right)=\phi_{1}\left(\mathrm{GP}W_{0}+\sum_{j}\phi_{2}\left(\mathrm{concat}\left(\mathrm{GP}W_{1},\Delta_{i,j},y_{j}\right)W_{2}\right)\right)W_{3}$$

(8) $$\Delta_{i,j}=\phi \left(MLP\left(v_{i}-v_{j}\right)\right)$$

while ${\mathrm{GP}}^{\prime }\in R^{B\times K\times 2}$ is the feature of the *i*th actor, *B* refers to batch size and *K* is the number of prediction modes, $\phi_{i}$ is layer normalization process, $W_{i}$ serves as weights, and $y_{j}$ is the *j*th lane node feature.This process serves as spatial distance-based attention and updates the goal area lane nodes’ features back to the actors, enhancing the model’s ability to capture complex driving behaviors.

This Region of Interest (ROI) filtering method allows us to precisely determine potential target points for each actor and predict their possible future interactions. This strategy significantly enhances the model’s ability to understand and predict actor behaviors in complex traffic scenarios.

Finally, we use the updated vehicle features ${\mathrm{GP}}^{\prime }$ as input to generate *K* confidence scores as matrix $C\in R^{B\times K}$. Both of them will be used in the decoding process to predict final future trajectories. Similar to LaneGCN, it can be generated simply by MLP:

(9) $$C=\mathrm{ReLU}(\mathrm{MLP}\left(\mathrm{LinearRes}\left({\mathrm{GP}}^{\prime }\right)\right))$$

**Figure 4:**
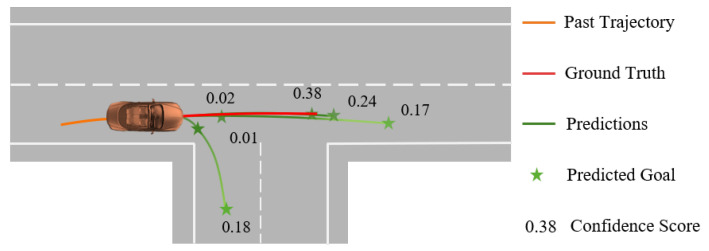
Predictive trajectory paths with associated confidence scores for an autonomous vehicle at an intersection. The encoding process captures past trajectories and environmental features for traffic scenario analysis.

(10) LinearRes()=GN(Linear(ReLU(GN(Linear())))

where GN( ) stands for GroupNorm. A dual-branch multimodal prediction architecture is employed, where one branch is responsible for estimating possible trajectories, and the other for assigning confidence scores to these trajectories. Figure 4 is a schematic diagram illustrating the model’s prediction of vehicle trajectories at an intersection and their associated confidence scores, where the green lines represent the predicted trajectories, the green stars indicate the predicted goals and the confidence scores for different trajectories are denoted by the numerical values along the paths. The following loss function is used to assess and optimize the accuracy of the predicted trajectories:

(11) $$L_{1}=\alpha_{1}L_{\mathrm{cls}\_\mathrm{end}}+\beta_{1}L_{\mathrm{reg}\_\mathrm{end}}+\rho_{1}L_{\mathrm{reg}\_\mathrm{mid}}$$

where $\alpha_{1}$, $\beta_{1}$, and $\rho_{1}$ serve as weights. By combining multi-target prediction and GoICrop technology, the model can precisely predict future trajectories among multiple possibilities.

### 4.4. Trajectory Decoding and Generation

The decoder of our model is designed to leverage the high-dimensional features extracted at earlier stages and generate accurate multimodal future trajectory predictions. This approach ensures the model’s capability to anticipate various potential pathways, enhancing the reliability of the predictions. To produce K∈N distinct predictions from the same input scenario, the model initially employs *K* learnable seed parameter matrices $Q_{i}\in R^{\left(d_{k},T\right)}$, where *T* denotes the prediction time steps, and i∈{1,…,K}. By replicating each $Q_{i}$ across the agent dimension, a new input tensor of dimension $R^{\left(d_{k},M,T\right)}$ is created, enabling the model to generate specific predicted trajectories for each agent at every time step, thereby effectively realizing diversified trajectory predictions for the same scenario.

The decoding process begins with handling the time dimension, employing a multi-head attention-based decoder (MABD) layer to process the encoder’s output ${\mathrm{GP}}^{\prime }$ and the encoded seed parameters $Q_{i}$. For *n* agents, this process can be denoted as

(12) $$H_{0}^{\prime }=\mathrm{MABD}\left(Q_{i},{\mathrm{GP}}_{n}^{\prime }\right)$$

(13) $$\mathrm{MABD}\left(C_{n},{\mathrm{GP}}_{n}^{\prime }\right)=\mathrm{LayerNorm}\left(H+rFFN\left(H\right)\right)$$

(14) $$H=\mathrm{LayerNorm}(H^{\prime }+\mathrm{MHSA}\left(H^{\prime },{\mathrm{GP}}_{n}^{\prime },{\mathrm{GP}}_{n}^{\prime }\right))$$

(15) $$H^{\prime }=\mathrm{LayerNorm}\left(\mathrm{MHSA}\left(C_{n}\right)\right)$$

where $H_{0}^{\prime }$ is the output tensor, MHSA represents the multi-head self-attention mechanism, and rFFN is a residual feed-forward network. These components work together, allowing the MABD layer to efficiently process and decode time-series data, generating precise future trajectory predictions for each agent.

To ensure social consistency among the set elements in future scenarios, it is essential to process each time slice of $H_{0}^{\prime }$. Specifically, for the agent state set $H_{0\tau }^{\prime }$ at some future time step τ, the decoder processes each element $h_{0\tau }^{\prime }$ using a multi-head attention block (MAB) layer.

(16) $$H_{0\tau }^{\prime }=\mathrm{MAB}\left(h_{0\tau }^{\prime }\right)$$

The decoder repeats these operations $L_{\mathrm{dec}}$ times, with each iteration that updates the output tensor $H_{0}^{\prime }$ progressively refining the prediction for each agent at future time steps. In decoding, different learnable seed parameters $Q_{i}$ and additional context information $m_{i}$ are used, repeating *c* times, resulting in a four-dimensional tensor $O\in R^{\left(d_{k}\times M\times T\times c\right)}$, containing all possible predictions. Finally, this output tensor can be element-wise processed through a ReLU activation function to produce the final output representation.

### 4.5. Training Details

The training process is divided into two stages: the target prediction stage and the regression stage. During the target prediction stage, we have adopted *K* mode endpoints’ estimations ${\mathrm{GP}}^{\prime }={\left\{g_{n,\mathrm{end}}^{k}\right\}}_{k\in [0,k-1]}$ and their confidence scores $C={\left\{c_{n,\mathrm{end}}^{k}\right\}}_{k\in [0,k-1]}$, where $g_{n,\mathrm{end}}^{k}$ is the *k*-th predicted goal coordinates and $c_{n,\mathrm{end}}^{k}$ is the *k*-th predicted goal confidence of the *n*-th actor. Our objective is to identify the positive target whose Euclidean distance to the ground truth trajectory endpoint is minimized. We employ a sum of classification and regression losses to train this stage. Given a predicted target, we aim to find the positive target with the minimum Euclidean distance to the ground truth trajectory endpoint. For classification, we utilize a maximum margin loss:

(17) $$L_{\mathrm{cls}\_\mathrm{end}}=\frac{1}{N(E-1)}\sum_{n=1}^{N}\sum_{k=\hat{k}}^{\hat{k}}\max\left(0,c_{k_{\left(n,\mathrm{end}\right)}}+\epsilon -{\hat{c}}_{k_{\left(n,\mathrm{end}\right)}}\right)$$

where *N* represents the total number of traffic participants, and ϵ=0.2 is the margin boundary. For the regression task, a smooth L1 loss is applied to all positive trajectory prediction steps:

(18) $$L_{\mathrm{reg}\_\mathrm{end}}=\frac{1}{N}\sum_{n=1}^{N}\mathrm{reg}\left(g_{{\hat{k}}_{n},\mathrm{end}}-a_{\left(n,\mathrm{end}\right)}^{*}\right)$$

where $a_{{\hat{k}}_{n},\mathrm{end}}$ is the ground truth BEV coordinates of the *n* actor’s trajectory endpoint, ${\hat{k}}_{n}$ denotes the *n* element, and reg() is the smooth L1 loss. Additionally, we attempt to incorporate a “single-target prediction” module at the midpoint of each trajectory to aggregate map features, assisting in the prediction of the endpoint target and overall trajectory. Similarly, for each actor, a residual MLP is applied to regress a middle target. The loss for this module is given by

(19) $$L_{\mathrm{reg}\_\mathrm{mid}}=\frac{1}{N}\sum_{n=1}^{N}\mathrm{reg}\left(g_{\left(n,\mathrm{mid}\right)}-a_{\left(n,\mathrm{mid}\right)}^{*}\right)$$

where $a_{\left(n,\mathrm{end}\right)}^{*}$ represents the ground truth BEV coordinates at the midpoint of the *n* actor’s trajectory. The total loss for the target prediction stage is

(20) $$L_{1}=\alpha_{1}L_{\mathrm{cls}\_\mathrm{end}}+\beta_{1}L_{\mathrm{reg}\_\mathrm{end}}+\rho_{1}L_{\mathrm{reg}\_\mathrm{mid}}$$

we set the weights of $\alpha_{1}$, $\beta_{1}$, and $\rho_{1}$ to 1, 0.2, and 0.1 in the experimental phase. In the regression stage, for classification, we employ a boundary loss $L_{\mathrm{cls}}$ similar to the one used in the target prediction stage. For regression tasks, the smooth L1 loss is similarly utilized and applied to all positive trajectory prediction steps:

(21) $$L_{\mathrm{reg}}=\frac{1}{NT}\sum_{n=1}^{N}\sum_{t=1}^{T}\mathrm{reg}\left(a_{\left(n,t\right)}^{\hat{k}}-a_{\left(n,t\right)}^{*}\right)$$

where $a_{\left(n,t\right)}^{\hat{k}}$ represents the predicted positive BEV coordinates of actor *n* at time step *t*, while $a_{\left(n,t\right)}^{*}$ represents a ground truth one.

Moreover, to emphasize the importance of the endpoint, we introduce a loss term that imposes a penalty at the endpoint:

(22) $$L_{\mathrm{end}}=\frac{1}{N}\sum_{n=1}^{N}\mathrm{reg}\left(a_{\left(n,\mathrm{end}\right)}^{\hat{k}}-a_{\left(n,\mathrm{end}\right)}^{*}\right)$$

The final training loss function is a weighted sum of these loss terms:

(23) $$L_{2}=\alpha_{2}L_{\mathrm{cls}}+\beta_{2}L_{\mathrm{reg}}+\rho_{2}L_{\mathrm{end}}$$

## 5. Performance Evaluation and Comparative Analysis

### 5.1. Experiment Setup

All the program tasks were conducted on Python 3.9, and the deep learning framework was based on PyTorch version 1.13. We train our model on a computer system equipped with an Intel(R) Xeon(R) Platinum 8358P CPU and an NVIDIA A40 GPU. Our predictive framework was evaluated on the extensive Argoverse motion prediction dataset, which provides trajectories for agent vehicles alongside high-definition map data. This dataset encompasses over 324,557 scenarios collected from Pittsburgh and Miami, segmented into training, validation, and test sets with 205,942, 39,472, and 78,143 samples, respectively. All training and validation scenarios consist of five-second sequences sampled at 10 Hz. In the trajectory prediction challenge hosted by Argoverse, the first 2 s of historical trajectory data are made available. Given the initial two-second observations, the Argoverse motion prediction challenge entails predicting the future three-second movement of agent vehicles. The dataset furnishes actor data as trajectories spanning 20 time steps; map data include a set of lane centerlines and their connectivity.

In addition to the Argoverse dataset, we also conducted evaluations on the nuScenes Prediction dataset [34], a self-driving car dataset collected in Boston and Singapore. It contains 1000 scenes, each lasting 20 s, with ground truth annotations and HD maps. Vehicles in nuScenes have manually annotated 3D bounding boxes, which are published at 2 Hz. The prediction task involves using the previous 2 s of object history and the map to predict the next 6 s. We employed the standard split from the nuScenes software (version 1.3) kit for our tests.

After data preprocessing, the relevant input features and desired outputs were extracted from both the training sets of Argoverse and nuScenes. The parameters of the network model were set according to the specifications outlined in Table 1.

### 5.2. Evaluation Metrics

This study evaluates experimental outcomes based on fundamental forecasting parameters and assessment metrics, focusing on unimodal (K = 1) and multimodal (K = 6) prediction outcomes. In instances where the model generates more than K trajectories, only the predictions with the top K probability scores are considered. The evaluation metrics include the minimum average displacement error (minADE), minimum final displacement error (minFDE), Brier minimum final displacement error (brierFDE), and miss rate (MR). The details of each metric are as follows.

Average displacement error (minADE) measures the average accuracy of predictions by calculating the average Euclidean distance between the ground truth trajectory and the best trajectory out of *K*-predicted trajectories. The formula for ADE is

(24) $$\mathrm{ADE}=\frac{1}{N}\sum_{i=1}^{N}\left(\frac{1}{T_{\mathrm{pred}}}\sum_{t=T+1}^{T+T_{\mathrm{pred}}}\sqrt{{\left(\hat{y}-y_{t}^{\mathrm{gt}}\right)}^{2}}\right)$$

Here, *N* is the number of predicted trajectories, $T_{\mathrm{pred}}$ is the prediction duration, *T* is the observation duration, $\hat{y}$ is the predicted position at time *t* which derives from *O* ($O={\left\{y_{n}^{k}\right\}}_{k\in [0,k-1]}$), and $y_{t}^{\mathrm{gt}}$ is the ground truth position at time *t*.

Final displacement error (minFDE) evaluates the accuracy of the predicted trajectory at the final moment of the prediction period by measuring the Euclidean distance between the last point of the predicted trajectory and the last point of the true trajectory. Its formula is

(25) $$\mathrm{FDE}=\frac{1}{N}\sum_{i=1}^{N}\sqrt{{\left({\hat{y}}_{\left(T+T_{\mathrm{pred}}\right)}-y_{\left(T+T_{\mathrm{pred}}\right)}^{\mathrm{gt}}\right)}^{2}}$$

The symbols used here carry the same meaning as those in the ADE formula. Brier minimum final displacement error (brier-minFDE) is similar to FDE but incorporates a penalty term related to the accuracy of the predicted probabilities in calculating the error. This metric considers not only the final displacement error of the prediction, but also the probability accuracy of the predicted trajectory. The miss rate (MissRateK,2) imposes penalties solely on predictions that deviate by more than 2 m from the ground truth. The offroad rate quantifies the proportion of predictions that fall outside the road boundaries.

### 5.3. Results and Ablation Studies

#### 5.3.1. Performance Comparison to Other Methods

We conducted an extensive comparison of our SRGAT model against a broader range of state-of-the-art trajectory prediction methods on the Argoverse motion prediction benchmark [2]. As presented in Table 2, SRGAT demonstrates superior performance over existing methods, notably TNT [22], LaneRCNN [35], LaneGCN [31], and the newly compared models in [23,25,36,37]. The detailed analysis reveals that SRGAT consistently achieves lower average offset error and higher long-term prediction accuracy, indicating its robustness in diverse traffic scenarios.

Notably, the comparison with LaneGCN, which serves as our primary benchmark, highlights the effectiveness of our approach. Our model achieves significant improvements of 15%, 22%, 21%, 15%, and 13% in minADE_6_, minFDE_6_, brierFDE_6_, minADE_1_, and minFDE_1_, respectively. These improvements can be attributed to the innovative use of the Social Relationship Graph Attention Network (SRGAT), which effectively captures dynamic interactions among agents in traffic, providing a more accurate prediction of their future trajectories. Our model’s profound comprehension of the dynamics between traffic participants and HD maps is facilitated by constructing a comprehensive map node graph coupled with a multi-layer graph neural network strategy. Leveraging HD map data alongside the Transformer network’s aptitude for identifying long-range dependencies, it can skillfully forecast a range of potential objectives and Points of Interest (POIs). Employing a dual-branch, multimodal prediction framework, SRGAT, not only generates multiple viable future pathways linked to these POIs, but also accurately evaluates their likelihood. This holistic integration of technologies ensures SRGAT achieves significant improvements in trajectory prediction accuracy compared to previous models, effectively enhancing our understanding and forecasting of complex traffic interactions.

Furthermore, we offer both quantitative metrics and qualitative insights to understand the model’s performance better. Through visual comparisons in specific scenarios, SRGAT not only accurately predicts trajectories, but also adapts to complex interactions, demonstrating its significant advantages over conventional models.

#### 5.3.2. Ablation Study

We conducted a detailed ablation study on the validation set to assess the impact of each component within our model. Taking the LaneGCN model as a baseline, we add other components progressively. Firstly, to enhance the model’s understanding of social interactions among traffic participants and avoid the inefficiencies observed during the inference process in graph neural networks (such as LaneGCN), we integrated an independent attention mechanism as the social interaction encoder in our model. In Table 3, ‘Social-En’ represents the social interaction encoder, and the ‘-’ symbol indicates its replacement with a three-layer FPN. Secondly, to better leverage road features and enhance the model’s understanding of the interaction between participants and the HD maps, we constructed a graph of map nodes and utilized a multi-step graph neural network to encode vectorized map information. Subsequently, we integrated the dynamic impact of participants on lanes with the real-time feedback of lanes on participant behavior using a Transformer Fusion Layer. These processes are denoted as Scene-En, with “-” indicating the substitution with feature extraction using a 32-layer CNN from rasterized map data. When combining the above two modules, we observe a performance improvement of over 54% on minFDE_6_, indicating the complementary effects of these modules and their importance in enhancing model performance. To improve training efficiency and model quality, we also employed learnable seed parameters, denoted as L-Seed. The introduction of seed parameters also makes an important contribution to further improving model performance.

#### 5.3.3. Qualitative Results

**Figure 5:**
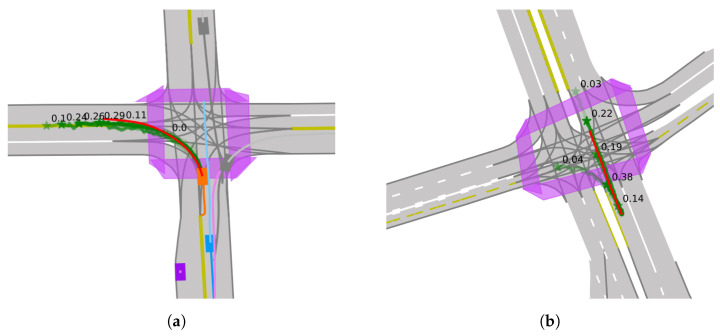

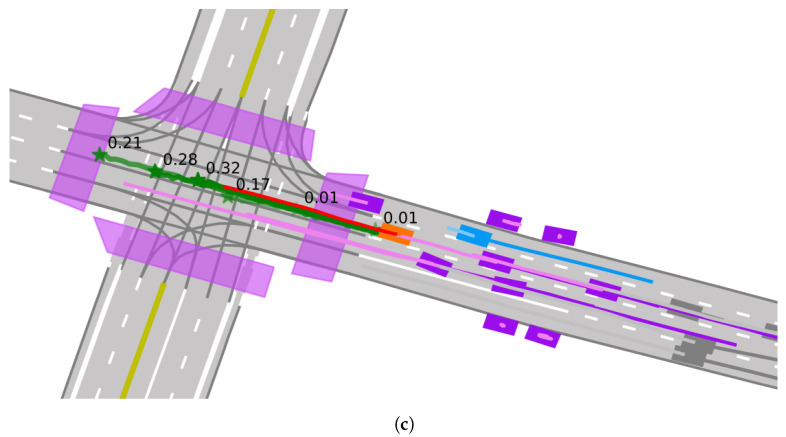
Qualitative results of SRGAT model. (**a**) Model predicts the vehicle’s left turn. (**b**) Model predicts straight movement. (**c**) Model utilizes traffic interactions for accurate trajectory predictions.

To better demonstrate the model’s effectiveness in complex traffic scenarios, we visualized the prediction results. As illustrated, orange represents the actual location of the target vehicle, blue signifies the ego vehicle, and purple indicates the relevant other traffic participants. The red line depicts the actual trajectory (ground truth) of the target vehicle, while the green lines represent the multimodal predictive trajectories generated by our model, green stars indicate the predicted goals, each with a corresponding confidence level. From Figure 5a,b, we can observe that the model made accurate predictions about the target vehicle’s direction of travel at the intersection. In Figure 5c, our model effectively utilized the interactions among surrounding traffic participants to generate accurate trajectory predictions for the target vehicle. These details demonstrate our model’s advanced capability in understanding and adapting to complex traffic scenarios, accurately predicting vehicle behaviors, and providing reasonable predictions among various potential behavioral choices. These results underscore the predictive power and accuracy of our model in complex traffic situations.

## 6. Conclusions

This article presents SRGAT, a cutting-edge trajectory prediction model for predicting vehicle trajectories in advanced autonomous driving applications, leveraging high-definition map data and vehicle dynamics. Its unique architecture, which combines a Transformer network with a dual-branch multimodal prediction mechanism, enables it to effectively capture complex traffic scenarios and predict future vehicle movements with high precision. The use of goal area estimation strengthens the model’s ability to generate muti-mode trajectories and support effective use of road context. The integration of map data enhances the model’s contextual understanding, while the attention mechanism and learnable seed parameters improve prediction diversity and training efficiency. Through comprehensive testing on the Argoverse dataset, our model demonstrates superior performance over existing methods. The results highlight SRGAT’s advancement in trajectory prediction, showcasing its enhanced accuracy, reliability, and efficiency in predicting traffic movements.

## Figures and Tables

**Table 1:** The configuration of model parameters.

Hyperparameter	Value
Learning Rate	$10^{-2},10^{-3},10^{-4},10^{-5}$
Epoch Number	50
Batch Size	64
Self-Attention Unit Number	128
Activation	ReLU
Number of attentions	4

**Table 2:** Results on Argoverse (upper set) and nuScenes (lower set) motion forecasting dataset. The “-” denotes that this result was not reported in their paper.

Method	brierFDE_6_	minFDE_6_	minFDE_1_	minADE_6_	minADE_1_
LaneRCNN [35]	2.14	1.45	3.69	0.90	1.68
TNT [22]	2.14	1.44	4.95	0.91	2.17
DenseTNT (MR) [38]	2.07	1.38	3.69	0.91	1.70
LaneGCN [31]	2.05	1.36	3.77	0.86	1.70
mmTransformer [39]	2.03	1.33	4.00	0.84	1.77
HOME [40]	-	1.45	3.73	0.94	1.73
GOHOME [41]	1.98	1.45	3.64	0.94	1.68
DenseTNT (FDE) [38]	1.97	1.28	3.63	0.85	1.67
TPCN [42]	1.92	1.24	3.48	0.81	1.57
GANet [25]	1.79	1.16	3.45	0.80	1.59
R-Pred [23]	1.77	1.12	3.47	0.76	1.58
ProphNet [36]	1.73	1.14	3.33	0.77	1.52
QCNet [37]	1.69	1.07	-	**0.73**	-
**Ours**	**1.62**	**1.05**	**3.25**	**0.73**	**1.45**
**Method**	**MinADE_5_**	**minADE_10_**	**MissRate_5,2_**	**MissRate_10,2_**	**Offroad Rate**
CoverNet [43]	1.96	1.48	0.67	-	-
Trajectron++ [44]	1.88	1.51	0.70	0.57	0.25
SG-Net [45]	1.86	1.40	0.67	0.52	0.04
MHA-JAM [17]	1.81	1.24	0.59	0.46	0.07
CXX [46]	1.63	1.29	0.69	0.60	0.08
P2T [47]	1.45	1.16	0.64	0.46	0.03
PGP [48]	1.30	1.00	0.61	0.37	0.03
**Ours**	**1.22**	**0.95**	**0.58**	**0.33**	**0.03**

**Table 3:** The ablation study of SRGAT model.

Scene-En	Social-En	L-Seed	ADE_6_	FDE_6_
✓	-	-	1.49	5.31
-	✓	-	1.41	5.10
-	-	✓	1.33	4.72
✓	✓	-	1.04	2.40
✓	✓	✓	0.83	1.30

## Data Availability

The data presented in this study are available on request from the corresponding author.
